# Can femoral head necrosis induced by steroid therapy in patients infected with coronaviruses be reversed?

**DOI:** 10.1038/s41413-020-00132-y

**Published:** 2021-01-08

**Authors:** Honglin Wang, Lei Niu

**Affiliations:** Department of Microscopic Orthopedics, The Hefei Second People’s Hospital & Hefei Affiliated Hospital of Anhui Medical University, 574 Changjiang East Road, Hefei, Anhui China

**Keywords:** Endocrine system and metabolic diseases, Diseases

Recently, we read with interest the findings of Zhang et al.^1^ of the long-term bone consequences in severe acute respiratory syndrome (SARS) patients. Their conclusion suggested that pulmonary damage and femoral head necrosis could be halted and reversed in SARS patients during the 15-year follow-up. These results have very important implications for the treatment and health utility evaluation of coronavirus disease 2019 (COVID-19). However, they did not explore the reasons for the reversal of femoral head necrosis after large doses of steroid pulse therapy, which may affect our understanding with regard to the treatment of COVID-19 patients.

The causes of femoral head necrosis in patients treated with steroid therapy are mainly the result of two risk factors, i.e., the infecting coronavirus and host hormones. First, we will discuss the possible potential mechanisms underlying femoral head necrosis caused by coronavirus infection. On the one hand, the virus causes an inflammatory response and stimulates the release of a large number of inflammatory cytokines, resulting in abnormal hemodynamics of the femoral head. On the other hand, hypoxemia causes an imbalance between the oxygen demand of the femoral head and the oxygen supply provided by the circulation.^2^ In addition, the expression of the angiotensin-converting enzyme 2 receptor in human vascular endothelial cells may be involved in the pathological process.^3^ Second, we will discuss possible reasons for steroid-induced femoral head necrosis. This phenomenon is related to the daily steroid doses, cumulative steroid dosages, and individual genetic backgrounds.^4^ In their study, only 2 of 15 SARS patients (i.e., coded as case 11 and case 14) reversed their association research circulation osseous (ARCO) stage,^1^ but the authors did not report the doses and duration of steroids administered to the two patients, nor did they analyze the associations of ARCO stage with steroid dose, steroid duration, and personal medical history. Therefore, we suspect that this was just an individual phenomenon and cannot support their conclusion that femoral head necrosis in patients treated with high doses of steroids is reversible.

Their conclusion should be interpreted with caution, and the treatment of COVID-19 patients should involve low doses of steroids to maintain the clinical efficacy of treatment while minimizing the risks.^5^ In addition, magnetic resonance imaging (MRI) is essential for the diagnosis of femoral head necrosis and has a high detection rate,^6^ suggesting that MRI should be performed routinely during the treatment of COVID-19 patients. Furthermore, COVID-19 patients should limit their intake of tobacco and alcohol, eat more fresh vegetables and fruits, control their diet to reduce their weight, and take calcium plus vitamin D (under the guidance of doctors).
